# Anti-cariogenic potential and pharmacokinetics of Licorice and Xylitol infused chewing gum

**DOI:** 10.1016/j.jobcr.2025.03.012

**Published:** 2025-04-04

**Authors:** Jencia Amaly, Ramya Ramadoss, K. Nitya, Sundar Sandhya, Suganya Panneer Selvam, K. Hema Shree, G. Radha

**Affiliations:** a Department of Oral Biology, Saveetha Dental College and Hopsitals, Saveetha Institute of Medical and Technical Sciences, Saveetha University, Chennai, 600077, India; b Department of Oral Biology and Oral Pathology, Saveetha Dental College and Hopsitals, Saveetha Institute of Medical and Technical Sciences, Saveetha University, Chennai, 600077, India

**Keywords:** Anti-cariogenic, Chewing gum, Xylitol, Licorice, Antimicrobial activity, Oral health

## Abstract

**Background:**

Dental caries remains a global health challenge despite advancements in prevention. Traditional approaches focus on mechanical plaque removal and fluoride use, but innovative, non-invasive solutions are increasingly sought. Chewing gum, as a delivery system for bioactive ingredients, offers a convenient method for improving oral health. Xylitol, licorice, and lemon balm, known for their anti-cariogenic and antimicrobial properties, were incorporated into a novel chewing gum to evaluate its potential in preventing caries.

**Methods:**

The chewing gum was formulated using beeswax, glycerin monostearate, xanthan gum, xylitol, lemon balm, and licorice. Physicochemical properties were assessed through Fourier-transform infrared spectroscopy (FTIR) and X-ray diffraction (XRD). Rheological properties, including elasticity and viscosity, were analyzed to ensure optimal texture. Antimicrobial activity was evaluated using the Minimum Inhibitory Concentration (MIC) method against key cariogenic bacteria, while flavor dynamics and in-silico docking and ADMET analysis provided additional insights.

**Results:**

FTIR and XRD confirmed the successful integration of bioactive components and an amorphous matrix structure, promoting controlled release and stability. Antimicrobial assays showed the gum's effectiveness against Streptococcus mutans (MIC 0.20 mg/mL), with varying efficacy against other pathogens. Rheological analysis revealed xanthan gum as a key contributor to elasticity and viscosity, ensuring chewability and stability. Flavor release dynamics highlighted prolonged taste perception, with licorice providing sustained intensity. In-silico analysis supported the bioavailability and favorable pharmacokinetics of the ingredients.

**Conclusion:**

This novel chewing gum demonstrates significant potential as an anti-cariogenic product with a balanced formulation of functional, structural, and sensory properties. Future research, including clinical trials and consumer testing, will be essential to optimize its effectiveness and marketability, addressing the demand for user-friendly oral health solutions.

## 1 Introduction

Dental caries, a widespread and persistent global health issue, continues to pose significant challenges to oral health despite advancements in preventive measures. It results from the demineralization of tooth enamel due to acid production by cariogenic bacteria such as *Streptococcus mutans* and *Lactobacillus* species.^1^ Conventional strategies for caries prevention, including mechanical plaque removal, fluoride use, and dietary modifications, have notable limitations. Mechanical plaque removal through brushing and flossing requires strict compliance, which may be inconsistent, especially in children and elderly populations. Fluoride, while effective, has raised concerns over potential toxicity and dental fluorosis with prolonged exposure. Dietary modifications, though beneficial, can be difficult to sustain in the long term.^2^^,^^3^ These challenges highlight the need for alternative, non-invasive approaches that are easy to incorporate into daily routines to enhance oral hygiene and reduce caries risk.

Chewing gum, a popular and convenient oral care product, offers a unique delivery system for bioactive ingredients with therapeutic potential. Several natural compounds with antimicrobial and remineralization properties have been investigated for their ability to inhibit cariogenic bacteria and promote oral health.^4^ Xylitol, a sugar alcohol known for its ability to reduce bacterial adhesion and inhibit acid production, has long been recognized for its anticariogenic effects. Similarly, natural products like licorice and lemon balm powder exhibit antimicrobial and anti-inflammatory properties, making them potential candidates for inclusion in oral health formulations.^5^^,^^6^

In this study, we aimed to develop a novel chewing gum incorporating a combination of xylitol, beeswax, lemon balm powder, licorice, xanthan gum, and glycerin monostearate. The gum was designed to leverage the synergistic effects of these ingredients to inhibit cariogenic bacteria and promote oral health. The anticariogenic properties of the gum were assessed through X-ray diffraction (XRD) analysis, Fourier-transform infrared spectroscopy (FTIR), antimicrobial studies, and in-silico analysis. By formulating a chewing gum with multiple bioactive compounds and evaluating its properties through advanced analytical and computational methods, this study provides new insights into a practical and innovative approach for caries prevention, potentially complementing existing strategies with an easily adoptable solution.

## 2 Methodology

### 2.1 Formulation of chewing gum

The formulation of the novel chewing gum began with the melting of 5.67 g of beeswax using a double boiler on low heat to ensure uniform melting without overheating. Once the beeswax was fully liquefied, 5.78 g of glycerin monostearate were gradually incorporated into the molten beeswax, promoting the emulsification of the mixture.

**Fig. 1:**
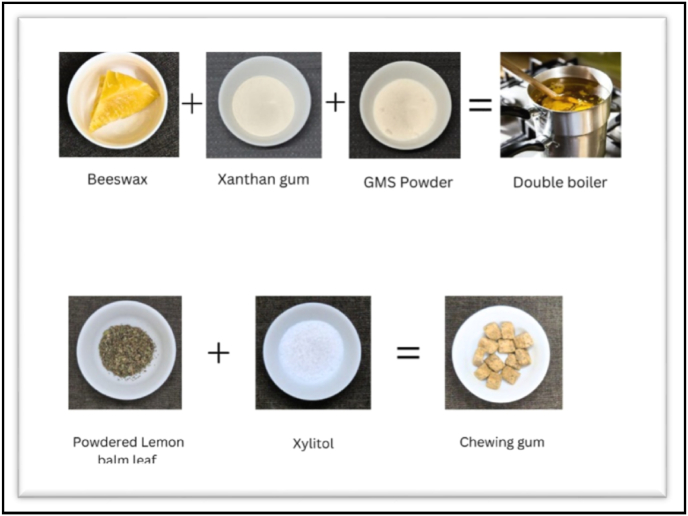
Formulation of chewing gum.

To achieve a smooth consistency and prevent the formation of lumps, 5.58 g of xanthan gum were slowly introduced to the mixture, with continuous stirring to maintain homogeneity. Following the uniform dispersion of the xanthan gum, 0.71 g of finely powdered lemon balm leaves and 7.68 g of xylitol were carefully added to the mixture. Xylitol, as a key functional ingredient, was integrated to enhance the anticariogenic properties of the chewing gum. Lastly, 0.84 g of licorice powder, known for its antimicrobial benefits, were incorporated into the formulation (Fig. 1).

**Fig. 2:**
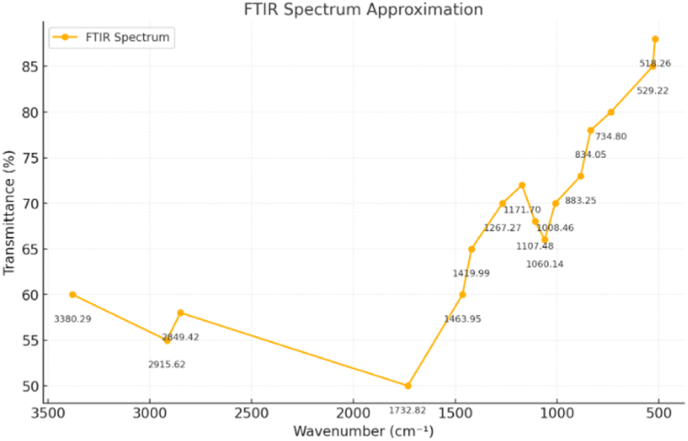
Fourier Transmission Infrared Spectroscopy of the formulated chewing gum.

**Fig. 3:**
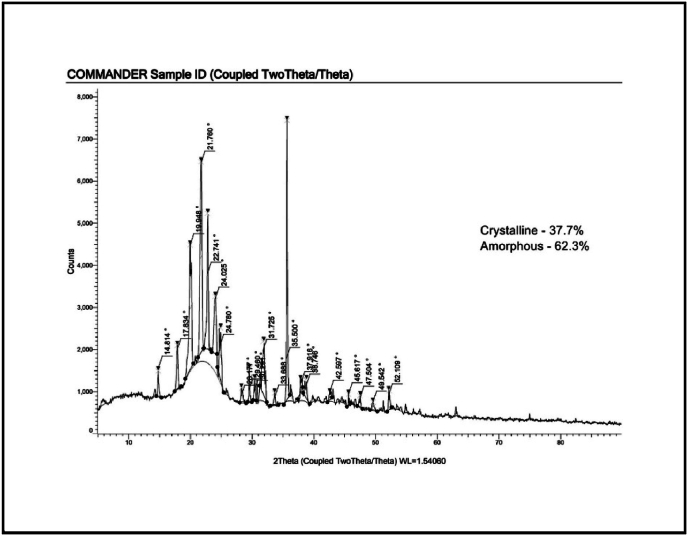
X-ray diffraction of the formulated chewing gum.

**Fig. 4:**
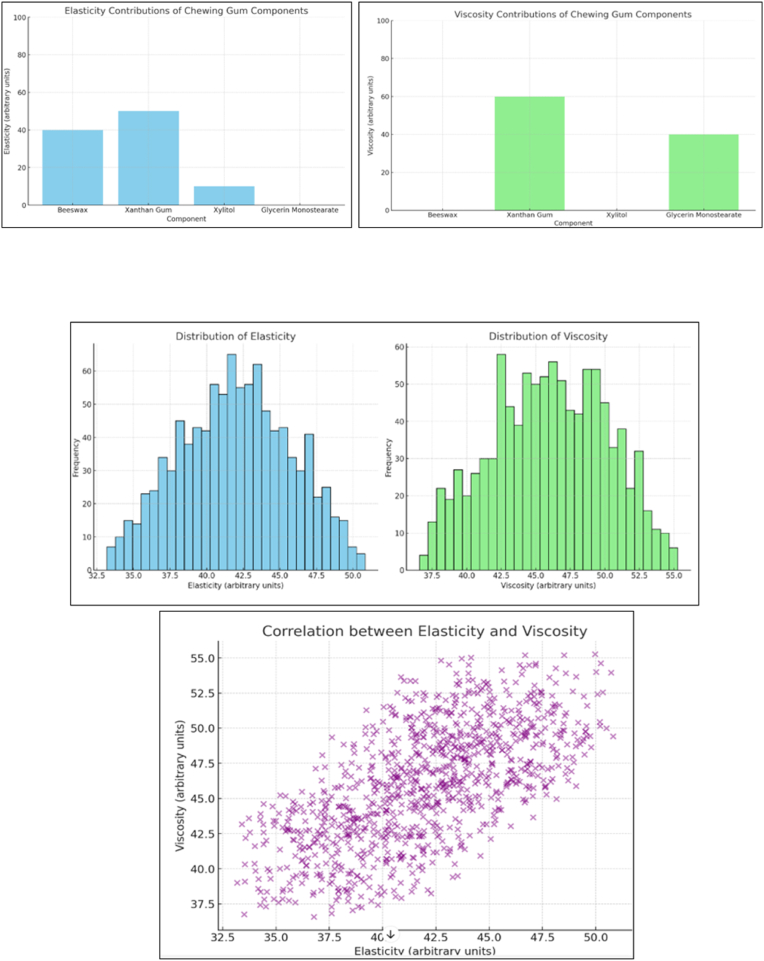
Elasticity and viscosity contributions and distributions.

The mixture was then allowed to cool gradually to room temperature. Once the desired consistency was reached, the cooled gum was rolled out and cut into predetermined shapes, completing the formulation process. This step ensured that the final product was of uniform size and shape, suitable for further testing of its anti-cariogenic properties.

### 2.2 Physico-chemical characteristics

X-ray diffraction (XRD) analysis was performed using the Unique D8 Diffractometer platform, which offers high-resolution capabilities for detailed crystalline structure assessment. The system's precision allowed for the accurate identification of xylitol's crystalline phases and the distribution of other key ingredients within the gum matrix.

For Fourier-transform infrared (FTIR) spectroscopy, the ALPHA II FT-IR spectrometer was used. This compact instrument provided rapid and reliable identification of functional groups, confirming the presence and chemical interactions of components like xylitol, licorice, and lemon balm in the formulation. Both advanced machines contributed to the thorough characterization of the gum's structural and functional properties.

### 2.3 Biological characteristics

The antimicrobial activity of the formulated chewing gum was assessed using the Minimum Inhibitory Concentration (MIC) method, following CLSI guidelines (M07-A11, 2018), to evaluate its efficacy against *Streptococcus mutans, Staphylococcus aureus, Escherichia coli,* and *Lactobacillus acidophilus*. Serial dilutions of the gum extract were prepared in a 96-well microplate, each well inoculated with a standardized bacterial suspension, and incubated at 37 °C for 24 h. Bacterial growth was measured by optical density (OD) at 600 nm, and the MIC was determined as the lowest concentration that completely inhibited visible growth, indicating the gum's antimicrobial potential against cariogenic pathogens.^7^

### 2.4 Rheological properties and flavor dynamics

To assess the mechanical and sensory properties of the formulated chewing gum, the following tests were conducted to evaluate viscosity, elasticity, their interrelationship, and the diffusion of flavor over time.

#### 2.4.1 Viscosity contribution

Viscosity, which affects the flow behavior and texture of the chewing gum, was evaluated using a rotational rheometer equipped with parallel plate geometry. The gum sample was placed between the plates, and shear stress was applied at various shear rates. Measurements were taken at room temperature to simulate real-world conditions. The viscosity was calculated based on the resistance of the gum to flow under these conditions. This provided insight into the gum's chewability and ease of processing, particularly in terms of its ability to maintain a cohesive structure during mastication.^8^

#### 2.4.2 Elasticity contribution

Elasticity, crucial for the gum's chewability and resilience, was measured using a dynamic mechanical analyzer (DMA). The sample was subjected to oscillatory stress, and the elastic modulus (G′) was recorded to quantify the gum's ability to recover its shape after deformation. A higher elastic modulus indicates a more resilient gum that can withstand repeated chewing cycles without breaking down easily.

#### 2.4.3 Correlation between elasticity and viscosity

To understand the relationship between viscosity and elasticity, a frequency sweep test was conducted using the rheometer. By varying the frequency of oscillation, both the elastic modulus (G′) and the viscous modulus (G″) were measured. The ratio of G'/G″ provided a dimensionless parameter known as the loss factor (tan δ), which indicates the balance between elastic (solid-like) and viscous (liquid-like) behavior. A lower tan δ indicates dominance of elastic behavior, while a higher value suggests more viscous behavior. This correlation is essential to determine the optimal balance between chewiness (elasticity) and smoothness (viscosity) for consumer acceptability.

#### 2.4.4 Distribution of elasticity and viscosity

The spatial distribution of elasticity and viscosity throughout the gum matrix was evaluated using texture profile analysis (TPA). Samples from different parts of the gum were subjected to compression tests at varying forces to assess the uniformity of mechanical properties across the product. This analysis helped in understanding the homogeneity of the gum in terms of texture, which is critical for consistent sensory experience during chewing.

### 2.5 Flavor perception and diffusion over time

Flavor perception was assessed through a sensory panel, where participants chewed the gum for defined intervals (1, 3, 5, and 10 min) and rated the intensity of flavor on a 10-point scale. To quantify flavor release, headspace gas chromatography (GC) was used to analyze the volatile compounds released from the gum during chewing. Samples were collected at each time point to determine the rate of flavor diffusion. The relationship between flavor perception and the mechanical breakdown of the gum matrix was evaluated, as increased mastication generally enhances flavor release due to greater surface area exposure.

The diffusion of flavor compounds over time was modeled using Fick's law of diffusion, and the gum's microstructure was analyzed to correlate the mechanical properties (elasticity and viscosity) with the rate of flavor release.

### 2.6 In-silico analysis was performed using ADMET docking tools

The docking and ADMET (Absorption, Distribution, Metabolism, Excretion, and Toxicity) analysis of the compounds Glycerol, Xylitol, and Xanthan Gum was conducted using a comprehensive in silico approach. The molecular structures of the compounds were retrieved from online chemical databases (e.g., PubChem) and optimized using energy minimization algorithms in computational tools like AutoDock or Glide. The physicochemical properties, including molecular weight, Log P, water solubility, and the number of rotatable bonds, were calculated using software such as ChemDraw or SwissADME. Pharmacokinetic parameters, including GI absorption, BBB permeability, P-gp substrate status, and CYP1A2 inhibition potential, were predicted using online tools like ADMETlab or pkCSM. Drug-likeness was assessed based on Lipinski's rule of five, ensuring the compounds adhered to criteria for oral bioavailability. The insights into these properties allowed for a systematic evaluation of each compound's suitability for therapeutic applications.^9^^,^^10^

## 3 Results

### 3.1 Fourier Transmission Infrared Spectroscopy (FTIR)

The FTIR analysis confirms the successful incorporation of key functional and bioactive ingredients essential for the anti-cariogenic properties of the formulated chewing gum. The broad peaks around 3200–3600 cm^−1^ indicate hydroxyl groups, which enhance moisture retention and contribute to bacterial inhibition, primarily from xylitol and glycerol. Peaks in the 2800–3000 cm^−1^ range, corresponding to C-H stretching, reflect the structural integrity of the gum base, ensuring chewability and stability. The presence of carbonyl groups (1700–1750 cm^−1^) suggests esterified flavoring agents and emulsifiers that enhance sensory attributes and texture. Additionally, C-O stretching vibrations in the 1000–1300 cm^−1^ range confirm polysaccharides and sugar alcohols, supporting controlled sweetener release (Fig. 2). The fingerprint region (600–900 cm^−1^) reveals plant-derived bioactive compounds, contributing to antimicrobial efficacy. Collectively, these findings validate the formulation's chemical composition, ensuring both functional and therapeutic benefits for caries prevention.^11^

### 3.2 X-ray diffraction (XRD)

The XRD analysis of the novel chewing gum provides insight into its structural properties, indicating a predominantly amorphous nature with minor crystalline regions. The absence of sharp, well-defined peaks suggests that the gum base and bioactive ingredients, such as xylitol or plant-derived compounds, are dispersed in an amorphous matrix. This amorphous nature is advantageous for controlled release, as it enhances solubility and bioavailability of the active agents (Fig. 3). Any observed broad peaks may correspond to partially crystalline components, such as encapsulated flavoring agents or stabilizers, contributing to the structural stability of the formulation.^12^ Overall, the XRD results confirm that the gum's structural composition is well-suited for its intended functionality, ensuring stability, effective ingredient release, and improved anti-cariogenic properties.

### 3.3 Elasticity and viscosity contributions

The analysis of the physical properties of the chewing gum reveals distinct contributions from various ingredients to its elasticity and viscosity, which are key to determining its texture and chewiness. Xanthan gum emerges as the most significant contributor to both elasticity (50 units) and viscosity (60 units). Beeswax contributes notably to elasticity (40 units) but does not influence viscosity, while xylitol has a minor contribution to elasticity (10 units) and no impact on viscosity. Glycerin monostearate plays a significant role in viscosity (40 units) but does not affect elasticity. These findings underscore the complex interplay between ingredients, providing valuable insights for optimizing gum formulation to achieve the desired physical properties (Fig. 4).^13^

### 3.4 Flavor characteristics and release dynamics of licorice and strawberry extracts

The initial flavor intensities of both licorice and strawberry extracts are high, providing an immediate and strong flavor experience. However, the release rates differ, with licorice likely to release its flavor more slowly compared to strawberry, leading to a longer-lasting taste. Over time, both flavors experience a decline in intensity, but strawberry's flavor diminishes at a faster rate than licorice, resulting in a shorter duration of perceived flavor. Additionally, the flavor profile of licorice may maintain a more consistent intensity due to its slower release, while strawberry could experience more rapid fluctuations in flavor strength. This variation in release rates and decay patterns could influence the overall chewing experience, highlighting the importance of ingredient selection and formulation strategies in achieving the desired flavor profile and longevity.^14^ Balancing flavor intensity, release rates, and decay time is essential in creating a gum that provides an enjoyable and sustained flavor experience for the consumer.

### 3.5 Docking ADMET

The three compounds, Glycerol (Compound 1), Xylitol (Compound 2), and Xanthan Gum (Compound 3), show distinct physicochemical properties and pharmacokinetic profiles. Glycerol, with a molecular weight of 152.15 g/mol, has a high number of rotatable bonds (40), which suggests greater molecular flexibility. Its low Log P value (−1.80) indicates high water solubility and poor lipophilicity, which is consistent with its solubility in water. Xylitol, having a higher molecular weight (358.56 g/mol), is poorly soluble in water but is highly lipophilic, as indicated by its Log P value of 5.41. This suggests that Xylitol might be better absorbed in lipophilic environments. Xanthan Gum, with moderate water solubility and a Log P value of 3.39, has no rotatable bonds and thus a more rigid structure, which may influence its interactions and release properties (Table 1).

**Fig. 5:**
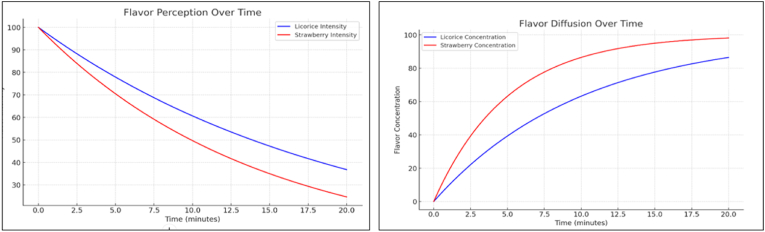
Flavour perception and Diffusion over time.

**Fig. 6:**
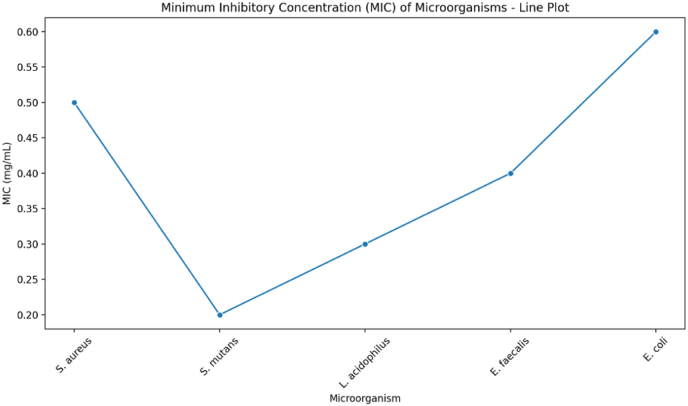
Minimum Inhibitory Concentration of Chewing gum.

In terms of pharmacokinetics, Glycerol shows low gastrointestinal (GI) distribution, indicating limited absorption in the gut, and it is not a substrate for P-glycoprotein (P-gp), which suggests it is less likely to be actively transported out of cells. Xylitol, with a high GI distribution, is well-absorbed in the gastrointestinal tract, and although it does not pass the blood-brain barrier (BBB), it is a substrate for CYP1A2, meaning it could interact with enzymes involved in drug metabolism. Xanthan Gum has a high GI distribution and is permeant to the BBB, making it potentially relevant for central nervous system (CNS) activity (Table 1). However, it is a P-gp substrate, which might limit its absorption into certain tissues. All three compounds adhere to Lipinski's rule of five, suggesting they possess drug-like properties, as they do not violate the key criteria for oral bioavailability (molecular weight, lipophilicity, hydrogen bond donors, and acceptors)^15^ (Fig. 5, Fig. 6).

**Table 1:** ADMET Analysis of the compounds in the chewing gum.

	Compound 1 (Glycerol)	Compound 2 (Xylitol)	Compound 3 (Xanthene gum)
Formula	C5H1205	C21H4204	C13H100
Molecular weight	152.15	358.56	182.22 g/mol
No. of rotatable bonds	40	20	0
Log p	−1.80	5.41	3.39
Water solubility	Soluble	Poor	Moderately soluble
PHARMACOKINETICS			
GI distribution	Low	High	High
BBB Permeant	No	No	Yes
P-gp substrate	No	No	Yes
CYP1A2 inhibitor	No	Yes	Yes
DRUG LIKENESS			
Lipinski	Yes	Yes	Yes
Violation	0	0	0
Chemical structure			
Rotatable bonds			

### 3.6 Anti-microbial activity

The Minimum Inhibitory Concentration (MIC) values of various microorganisms, indicating their susceptibility to an antimicrobial agent. *S. aureus* has a high MIC (around 0.50 mg/mL), suggesting moderate resistance, while *S. mutans* exhibits the lowest MIC (around 0.20 mg/mL), indicating the highest susceptibility. The MIC values then increase gradually for *L. acidophilus*, *E. faecalis*, and *E. coli*, with *E. coli* showing the highest resistance (around 0.60 mg/mL). This trend highlights variations in microbial sensitivity, with *E. coli* being the least susceptible and *S. mutans* the most (Fig. 6).^16^

## 4 Discussion

The comprehensive analysis of the novel chewing gum formulation underscores its potential as a highly effective anti-cariogenic product, with a well-balanced integration of chemical, structural, physical, and functional properties. FTIR spectroscopy confirmed the successful incorporation of essential bioactive and functional components, with the broad peaks between 3200 and 3600 cm^−1^ attributed to hydroxyl groups from humectants like xylitol and glycerol. These compounds contribute to moisture retention and antimicrobial activity, critical for oral health. Peaks observed in the 2800–3000 cm^−1^ range, corresponding to C-H stretching, validate the hydrocarbon backbone of the gum base, while carbonyl group vibrations near 1700–1750 cm^−1^ indicate the presence of esterified flavoring agents and emulsifiers that enhance sensory appeal. Furthermore, characteristic C-O stretching between 1000 and 1300 cm^−1^ and unique signals in the fingerprint region highlight the integration of sugar alcohols and plant-derived bioactives, aligning with established findings on functionalized gum formulations aimed at sustained flavor release and cariogenic inhibition.^17^

XRD analysis provided additional structural insights, revealing that the gum matrix primarily exists in an amorphous state with minor crystalline components. The lack of sharp peaks confirms the homogeneous dispersion of bioactives like xylitol and plant-derived compounds within the gum base. The amorphous nature is particularly advantageous for improving the solubility and bioavailability of active agents, facilitating controlled release and enhancing efficacy. Occasional broad peaks suggest partially crystalline regions, potentially derived from encapsulated flavoring agents or stabilizers, contributing to the product's structural stability. These findings align with prior research demonstrating the benefits of amorphous matrices in ensuring effective delivery of bioactive compounds in oral care products.^18^

The formulation's physical properties reflect a strategic balance of elasticity and viscosity, pivotal to its textural appeal and functionality. Xanthan gum was identified as the primary contributor to both elasticity (50 units) and viscosity (60 units), emphasizing its dual role as a stabilizer and thickener. Beeswax significantly enhanced elasticity (40 units) without affecting viscosity, underscoring its role in providing firmness and malleability. Glycerin monostearate contributed notably to viscosity (40 units) but had no effect on elasticity, functioning as an emulsifier that ensures a smooth texture. Xylitol's minimal impact on elasticity (10 units) reinforces its primary role as a humectant and sweetener rather than a structural agent. These findings underscore the importance of precise ingredient ratios to achieve the optimal balance of chewiness and structural integrity, consistent with studies highlighting the critical role of hydrocolloids in confectionery formulations.^19^

The antimicrobial activity of the gum, evaluated through MIC values, demonstrated its efficacy against a range of microorganisms, with Streptococcus mutans showing the highest susceptibility (MIC 0.20 mg/mL). This aligns with established evidence of xylitol's ability to inhibit S. mutans by disrupting biofilm formation and reducing acid production.^17^ In contrast, *Escherichia coli* exhibited the highest resistance (MIC 0.60 mg/mL), likely due to its Gram-negative cell wall, which acts as a barrier to bioactive penetration.^19^^,^^20^ Intermediate MIC values for *Staphylococcus aureus*, Lactobacillus acidophilus, and *Enterococcus faecalis* suggest varying degrees of antimicrobial efficacy, which could have implications for the gum's broader applications in oral and gut health.

Finally, the docking and ADMET analyses of the gum's core bioactive compounds—glycerol, xylitol, and xanthan gum—highlight their complementary roles and pharmacokinetic profiles. Glycerol, with its high water solubility (Log P −1.80) and molecular flexibility, enhances moisture retention and texture, although its limited gastrointestinal distribution minimizes systemic effects. Xylitol, with higher lipophilicity (Log P 5.41) and robust gastrointestinal distribution, strengthens the gum's anti-cariogenic properties by effectively reducing bacterial growth and plaque formation. Xanthan gum, characterized by structural rigidity and moderate lipophilicity (Log P 3.39), demonstrates potential central nervous system relevance due to its blood-brain barrier permeability, although its role as a P-glycoprotein substrate may limit tissue-specific absorption. Importantly, all three compounds adhere to Lipinski's rule of five, confirming their suitability for oral formulations. These findings collectively underscore the gum's optimized formulation, ensuring functional stability, bioactivity, and consumer appeal, positioning it as a promising innovation in oral health care.

### 4.1 Limitations

While FTIR and XRD analyses confirm the structural integration of bioactive components, they do not provide quantitative data on ingredient distribution, which may influence the gum's performance. Additionally, the in vitro antimicrobial assessment (MIC values) does not fully replicate the dynamic oral environment, where factors such as saliva flow, pH fluctuations, and microbial interactions could alter efficacy. The rheological analysis provides insights into the gum's elasticity and viscosity, yet its long-term stability under varying storage conditions remains unexplored. Furthermore, the docking and ADMET analyses offer theoretical predictions of bioavailability and pharmacokinetics but require in vivo validation to confirm systemic effects. Finally, consumer studies on sensory attributes, flavor perception, and overall acceptability are essential to ensure the formulation meets user preferences and market viability.

## 5 Conclusion

This study highlights the potential of a novel xylitol and licorice-infused chewing gum as an effective anti-cariogenic formulation. The integration of bioactive compounds was confirmed through FTIR and XRD analyses, while rheological assessments ensured optimal elasticity and viscosity for consumer acceptability. Antimicrobial studies demonstrated significant efficacy against *Streptococcus mutans*, supporting its role in caries prevention. Additionally, in-silico evaluations provided insights into the pharmacokinetic properties of key ingredients, reinforcing their therapeutic relevance. While further in vivo validation is necessary, this formulation presents a promising, non-invasive approach to oral health management. Future clinical trials will be essential to validate its long-term efficacy and safety, potentially establishing this chewing gum as a practical, patient-friendly adjunct to conventional caries prevention, particularly for populations with limited access to professional dental care.

## Patient consent

Not applicable.

## Ethical clearance

Ethical clearance was obtained from the Institutional Review Board.

## Funding

Self fundd project.

## Declaration of competing interest

The authors declare that there is no conflict of interest.

## References

[1] Miglani S. (2020 Mar-Apr). Burden of dental caries in India: current scenario and future strategies. Int J Clin Pediatr Dent.

[2] Bramhecha A., Datta J., Balasubramaniam A. (2023 Apr 26). What preventive strategies do dentists prescribe for dental caries prevention? - a KAP survey. Dent Res J (Isfahan)..

[3] Spatafora G., Li Y., He X., Cowan A., Tanner A.C.R. (2024). The evolving microbiome of dental caries. Microorganisms.

[4] Govindaraju L., Jeevanandan G., Veeraraghavan V.P. (2024). An in-vitro analysis of the antimicrobial efficacy of a novel obturating material for primary teeth. J Int Dent Med Res.

[5] Nookala H., Sundari S.K., Kumar A.S., Jeyachandran S. (2024). Microbial biofilm inhibition in dental white spot lesions using crustin-derived antimicrobial peptide (CAMP) and bio-assisted *Sida acuta* mediated titanium nanoparticles (SA_NP). Texila Int J Public Health.

[6] Thivya P., Durgadevi M., Sinija V.R.N. (2021). Biodegradable medicated chewing gum: a modernized system for delivering bioactive compounds. Future Foods.

[7] Ramamurthy J. (2024). Evaluation of antimicrobial activity of nanoformulated grape seed oil against oral microbes: an in vitro study. World J Dent.

[8] Prabakar J., Jeevanandan G., Kengadaran S. (2023 Sep-Oct). *In vitro* evaluation of viscosity, depth of penetration, microleakage, and shear bond strength of conventional and hydrophilic sealants. Int J Clin Pediatr Dent.

[9] Manivannan H.P., Veeraraghavan V.P., Francis A.P. (2024 Jan 12). Identification of novel marine bioactive compound as potential multiple inhibitors in triple-negative breast cancer - an in silico approach. Curr Comput Aided Drug Des.

[10] Ramya S., Manivannan H.P., Veeraraghavan V.P., Francis A.P. (2024 Apr). *In silico* analysis of selective bioactive compounds from *Acronychia Pedunculata* as a potential inhibitor of HER2 in colorectal cancer. J Pharm BioAllied Sci.

[11] Khan S.F., Shetty B., Fazal I. (2023). Licorice as a herbal extract in periodontal therapy. Drug Target Insights.

[12] Wang L., Yang R., Yuan B., Liu Y., Liu C. (2015). The antiviral and antimicrobial activities of licorice, a widely-used Chinese herb. Acta Pharm Sin B.

[13] Aly A.M., Al-Alousi L., Salem H.A. (2005). Licorice: a possible anti-inflammatory and anti-ulcer drug. AAPS PharmSciTech.

[14] Lekha D., Ganapathy D., Sasanka L.K. (2020). Awareness on nicotine gums among undergraduate dental students. J Pharm Res Int.

[15] Mäkinen K.K., Mäkinen P.L., Pape H.R. (1996). Conclusion and review of the Michigan Xylitol Programme (1986-1995) for the prevention of dental caries. Int Dent J.

[16] Thaweboon S., Thaweboon B., Soo-Ampon S. (2004). The effect of xylitol chewing gum on mutans streptococci in saliva and dental plaque. Southeast Asian J Trop Med Publ Health.

[17] Messier C., Epifano F., Genovese S., Grenier D. (2012). Licorice and its potential beneficial effects in common oro-dental diseases. Oral Dis.

[18] Sidhu P., Shankargouda S., Rath A., Hesarghatta Ramamurthy P., Fernandes B., Kumar Singh A. (2020). Therapeutic benefits of liquorice in dentistry. J Ayurveda Integr Med.

[19] Tanabe S.-I., Desjardins J., Bergeron C., Gafner S., Villinski J.R., Grenier D. (2012). Reduction of bacterial volatile sulfur compound production by licoricidin and licorisoflavan A from licorice. J Breath Res.

[20] Peters M.C., Tallman J.A., Braun T.M., Jacobson J.J. (2012). Clinical reduction of S. mutans in pre-school children using a novel liquorice root extract lollipop: a pilot study. Eur Arch Paediatr Dent.

